# Aquaporin 4 Mediates the Effect of Iron Overload on Hydrocephalus After Intraventricular Hemorrhage

**DOI:** 10.1007/s12028-023-01746-w

**Published:** 2023-05-19

**Authors:** Ying Li, Ding Nan, Ran Liu, Jieyu Li, Zhuangzhuang Zhang, Jianwen Deng, Yang Zhang, Ziguang Yan, Chao Hou, Ensheng Yao, Weiping Sun, Zhaoxia Wang, Yining Huang

**Affiliations:** 1 Department of Neurology, Peking University First Hospital, 8 Xishiku Street, Xicheng District, Beijing, 100034 China; 2 Beijing Key Laboratory of Neurovascular Disease Discovery, Beijing, China; 3 Department of Hyperbaric Oxygen, Beijing Chaoyang Hospital, Capital Medical University, Beijing, China; 4 Department of Neurosurgery, Peking University First Hospital, Beijing, China; 5 Department of Interventional Radiology and Vascular Surgery, Peking University First Hospital, Beijing, China; 6 Department of Radiology, Peking University First Hospital, Beijing, China; 7 Department of Neurology, First Affiliated Hospital, School of Medicine, Shihezi University, Xinjiang, China

**Keywords:** Intraventricular hemorrhage, Hydrocephalus, Iron, Aquaporin 4

## Abstract

**Background:**

Iron overload plays an important role in hydrocephalus development following intraventricular hemorrhage (IVH). Aquaporin 4 (AQP4) participates in the balance of cerebrospinal fluid secretion and absorption. The current study investigated the role of AQP4 in the formation of hydrocephalus caused by iron overload after IVH.

**Methods:**

There were three parts to this study. First, Sprague–Dawley rats received an intraventricular injection of 100 µl autologous blood or saline control. Second, rats had IVH and were treated with deferoxamine (DFX), an iron chelator, or vehicle. Third, rats had IVH and were treated with 2-(nicotinamide)-1,3,4-thiadiazole (TGN-020), a specific AQP4 inhibitor, or vehicle. Rats underwent T2-weighted and T2* gradient-echo magnetic resonance imaging to assess lateral ventricular volume and intraventricular iron deposition at 7, 14, and 28 days after intraventricular injection and were then euthanized. Real-time quantitative polymerase chain reaction, western blot analysis, and immunofluorescence analyses were conducted on the rat brains to evaluate the expression of AQP4 at different time points. Hematoxylin and eosin–stained brain sections were obtained to assess the ventricular wall damage on day 28.

**Results:**

Intraventricular injection of autologous blood caused a significant ventricular dilatation, iron deposition, and ventricular wall damage. There was increased AQP4 mRNA and protein expression in the periventricular tissue in IVH rats through day 7 to day 28. The DFX treatment group had a lower lateral ventricular volume and less intraventricular iron deposition and ventricular wall damage than the vehicle-treated group after IVH. The expression of AQP4 protein in periventricular tissue was also inhibited by DFX on days 14 and 28 after IVH. The use of TGN-020 attenuated hydrocephalus development after IVH and inhibited the expression of AQP4 protein in the periventricular tissue between day 14 and day 28 without a significant effect on intraventricular iron deposition or ventricular wall damage.

**Conclusions:**

AQP4 located in the periventricular area mediated the effect of iron overload on hydrocephalus after IVH.

**Supplementary Information:**

The online version contains supplementary material available at 10.1007/s12028-023-01746-w.

## Introduction

Intraventricular hemorrhage (IVH) often occurs in patients with intracerebral hemorrhage (ICH) or subarachnoid hemorrhage (SAH) and is associated with increased morbidity and mortality [1, 2]. Hydrocephalus, a disorder characterized by cerebrospinal fluid (CSF) accumulation in the ventricular system and subarachnoid space, develops in 50–67% of patients with IVH and is recognized as a predictor of poor outcome [1, 2]. The degree of ventricular dilatation is closely related to mortality [1, 2]. Hydrocephalus may also cause cognitive impairment and motor dysfunction [3].

However, the underlying mechanisms of IVH-induced hydrocephalus are not fully understood. Many studies have focused on the effects of neurotoxic compounds released from blood [4–6]. Iron overload is considered to play a key role in the development of hydrocephalus after IVH, which is supported by the inhibition of ventricular dilatation with the use of deferoxamine (DFX), an iron chelator [5, 6]. But how iron impacts the balance between CSF secretion and absorption remains unclear. Previous studies suggested that inflammatory pathways, scarring of the CSF outflow, and ependymal cilia injury may contribute to iron-related hydrocephalus [7–10].

Aquaporin 4 (AQP4) is the main water channel protein in the central nervous system [11–14]. The expression of AQP4 in ependymal cells and subependymal astrocytes is an important determinant of CSF flow across the ventricle–brain interface and participates in the production of CSF in physiological conditions [11]. Several studies have found an upregulated expression of AQP4 after experimental IVH [15, 16]. But the role of AQP4 in posthemorrhagic hydrocephalus development and the association between AQP4 and iron overload are not clear. We speculated that AQP4 might participate in the formation of hydrocephalus caused by iron overload following IVH.

The current study aimed to test our hypothesis in a rat model of IVH established by injection of autologous blood. We investigated the changes in ventricular volume, iron deposition, and ventricular wall damage, as well as AQP4 expression, at different time points after IVH. Then we tested the effects of DFX and 2-(nicotinamide)-1,3,4-thiadiazole (TGN-020), a specific AQP4 inhibitor, on ventricular volume, iron deposition, ventricular wall damage, and AQP4 expression.

## Methods

### Animal Preparation and Intraventricular Injection

The protocol for the animal model was approved by the Peking University First Hospital (Beijing, China) Committee on the Use and Care of Animals. The studies were in accordance with the Guide for the Care and Use of Laboratory Animals. All efforts were made to minimize the number of animals used and their suffering. A total of 102 adult male Sprague–Dawley rats (3 to 4 months old; provided by SPF Beijing Biotechnology) weighing 250–350 g were used in this study. Animals were housed at a specific pathogen-free facility with a 12-h light/dark cycle and free access to food and water. Animals were anesthetized with pentobarbital (50 mg/kg intraperitoneally). Core body temperature was maintained at 37.5 °C with a feedback-controlled heating pad. The right femoral artery was catheterized for blood collection. Rats were then placed in a stereotactic frame (World Precision Instruments, Sarasota, FL). A cranial burr hole (1 mm) was drilled and a 26-gauge needle was inserted stereotactically into the right lateral ventricle (coordinates: 0.6 mm posterior, 4.5 mm ventral, and 1.6 mm lateral to the bregma) [4, 5]. Either 100 µL of autologous arterial blood or 100 µL of saline was infused at a rate of 10 µL/min using a micro infusion pump (WPI, Sarasota, FL). The needle was removed 5 min after injection, the burr hole was filled with bone wax, and the skin incision was closed with sutures [4, 5].

DFX (500 mg per bottle; Novartis Pharma GmbH, Switzerland) was diluted with 5 mL of normal saline to prepare the final working concentration of 100 mg/mL for intraperitoneal injection of 100 mg/kg per animal [5–7]. TGN-020 (Sigma-Aldrich, USA) was diluted in dimethyl sulfoxide (DMSO) (Sigma-Aldrich, USA) to make a 10 mM stock solution. The concentration of DMSO used for intraperitoneal injection was adjusted at 0.1% for an injection of 50 mg/kg per animal [17].

### Experimental Groups

There were three parts to this study. In the first part, 30 male rats randomly received an injection of 100 µL of autologous blood or saline into the right lateral ventricle. Rats (IVH, *n* = 5 per time point; saline control, *n* = 5 per time point) received magnetic resonance imaging (MRI) on days 7, 14, and 28 and then were euthanized. The brains were used for histological examination, real-time quantitative polymerase chain reaction, and western blot analysis. In the second part, 36 male rats received an injection of 100 µL of autologous blood into the right lateral ventricle and randomly received DFX (100 mg/kg intraperitoneally, *n* = 6 per time point) or vehicle (same volume of saline, *n* = 6 per time point) treatment at 3 h after IVH and then every 12 h for 7 days. The rats underwent MRI scans on days 7, 14, and 28 and were then euthanized for brain histology. In the third part of the study, 36 male rats received an injection of 100 µL of autologous blood into the right lateral ventricle and were randomly assigned to receive TGN-020 (50 mg/kg intraperitoneally, *n* = 6 per time point) or vehicle (same volume of DMSO, *n* = 6 per time point) treatment after IVH every 24 h until euthanized. The rats received an MRI scan on days 7, 14, and 28, and their brains were used for histology.

### Magnetic Resonance Imaging

Imaging was performed in a 7.0-T Varian magnetic resonance scanner (Agilent, USA). Rats were anesthetized with a 2% isoflurane/air mixture throughout the MRI examination. T2-weighted (repetition time/echo time [TR/TE] = 3500/72 ms) and T2* gradient-echo sequences (TR/TE = 126.78/4.55 ms) were performed 7, 14, and 28 days after IVH onset or saline injection. A total of 15 coronal slices (1 mm thick) with a view field of 35 × 35 mm were taken to cover the entire axis of the lateral ventricles. All image analysis was performed using Image J version 1.45 software (National Institutes of Health, Bethesda, MD). Lateral ventricular volumes were calculated from the T2 images. Bilateral ventricles were outlined, and the areas were measured. Ventricular volume was calculated by summing the bilateral ventricular area in each slice and multiplying by slice thickness [4, 5]. The volume of intraventricular hypointensity, representing hemorrhage and iron accumulation, was calculated from T2* MRI using the same method as for ventricular volume [4, 5]. All the measurements were conducted by a blinded researcher.

### Real-Time Quantitative Polymerase Chain Reaction

Periventricular brain tissue (~ 1-mm-thick brain tissue around the ventricle) total RNA was isolated using Trizol reagent (INVITROGEN, USA), following the manufacturer’s protocol. Next, cDNA synthesis was performed using TransScript First-Strand cDNA Synthesis SuperMix (TransGen Biotech, China). Relative mRNA expression levels of the genes studied were quantified using real-time quantitative polymerase chain reaction analysis with ABI PRISM 7500 qPCR (Applied-Biosystems, USA), SYBR Green Master mix (Applied-Biosystems, USA), and the thermocycler conditions recommended by the manufacturer. The following primers were used:

- AQP4 forward primer: 5′GGTGGGAGGATTGGGAGTC3′
- AQP4 reverse primer: 5′CAGCGCCTA-TGATTGGTC3′
- β-actin forward primer: 5′AACCCTAAGGCCAACCGTGAAA-AG3′
- β-actin reverse primer: 5′TCATGAGGTGTCTGTCAGGT3′

All samples were performed in triplicate. The mRNA level of AQP4 was normalized to β-actin and calculated using the 2^−△△Ct^ method.

### Western Blot Analysis

Periventricular brain tissue (~ 1-mm-thick brain tissue around the ventricle) was sampled. The primary antibodies were anti-AQP4 antibody (1:2000 dilution, Proteintech Biotechnology, USA) and mouse anti-β-actin antibody (1:2000 dilution, ZSGB-BIO, China). The secondary antibodies were goat anti-rabbit (1:5000, ZSGB-BIO, China) and goat anti-mouse (1:5000, ZSGB-BIO, China). β-actin was applied as the control. The relative densities of bands were analyzed with Image J version 1.45 software (National Institutes of Health, Bethesda, MD).

### Immunofluorescence Analysis

Slices (10-μm thick) were incubated with 0.3% Triton-100 for 20 min and blocked with goat serum for 60 min. Next, the slices were incubated with rabbit anti-AQP4 antibody (1: 100 dilution, Proteintech Biotechnology, USA) overnight at 4 °C. After being rinsed in phosphate-buffered saline, slices were incubated with secondary antibodies (FITC labeled, 1: 200 dilution, ZSGB-BIO, China) for 60 min at room temperature in the dark. Finally, the slices were rinsed in phosphate-buffered saline and incubated with 4′,6-diamidino-2-phenylindole (ZSGB-BIO, China) for nuclear staining. The slices were imaged using a fluorescence microscope (Nikon, Japan) with × 20 magnification.

### Measurement of Ventricular Wall Damage

Hematoxylin and eosin (H&E)-stained brain sections were obtained for the rats on day 28. The length of the ependyma that was disrupted or detached from the periventricular parenchyma was measured, as well as the total bilateral ventricle wall length. The degree of ventricle wall damage was calculated as a percentage by dividing the length of disruption by the total ventricular surface perimeter [18]. The analyses were performed by a blinded observer using Image J software.

### Statistical Analysis

Values are given as mean ± standard error of mean (SEM). Student’s *t*-test was used to analyze data in accordance with normal distribution, and the Mann–Whitney *U*-test was used for the data inconsistent with normal distribution. The false discovery rate was used to correct for multiple comparisons. Differences were considered significant at *p* < 0.05. All statistical analyses were conducted using SPSS version 23.0 (IBM Corp., Armonk, NY).

## Results

### IVH Induces Ventricular Dilation, Iron Overload, and Ventricular Wall Damage

**Fig. 1:**
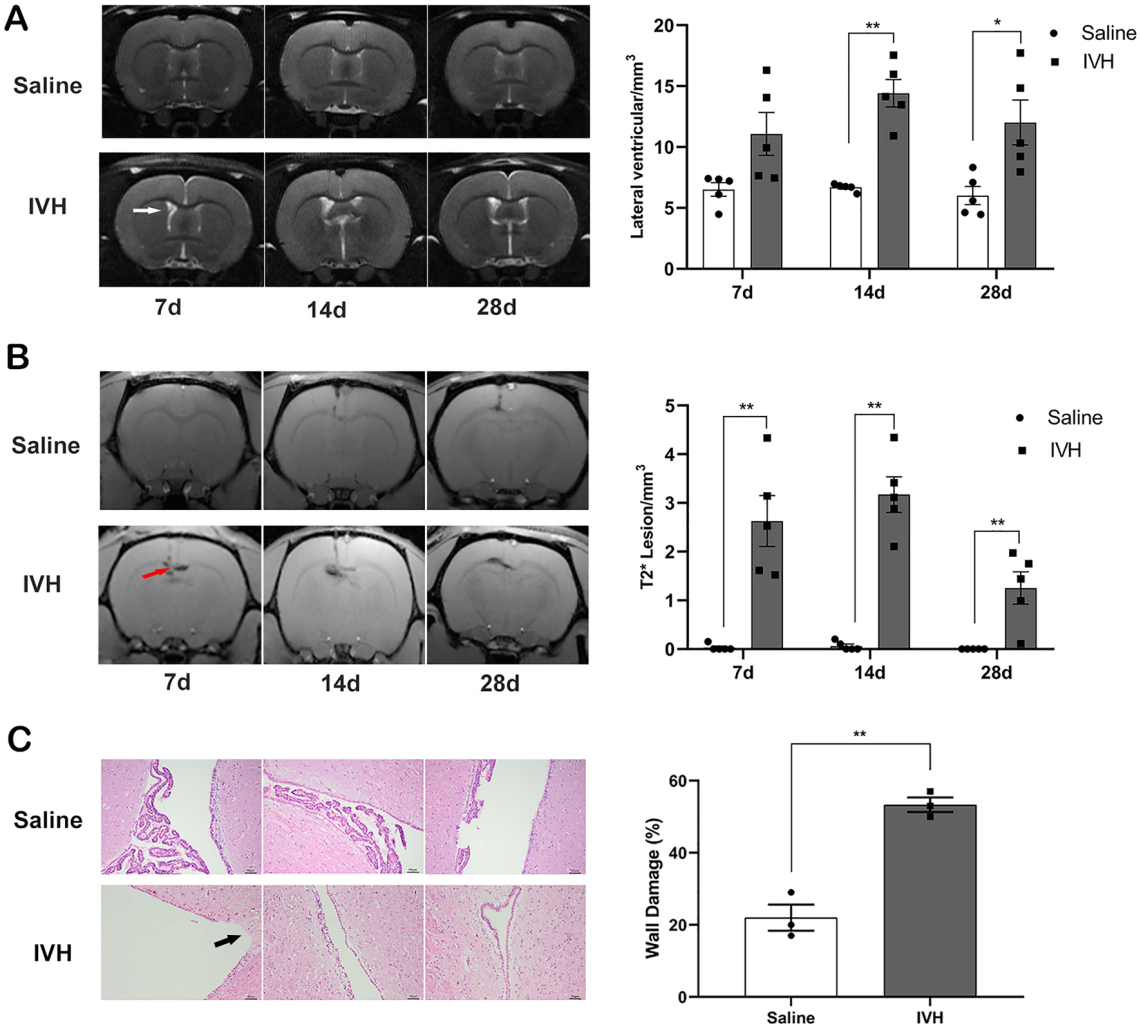
Intraventricular hemorrhage (IVH) induces ventricular dilation, intraventricular iron deposition, and ventricular wall damage. **a** Representative T2-weighted magnetic resonance imaging (MRI) scans (coronal brain sections) and quantification of ventricular volume at 7, 14, and 28 days after intraventricular injection of saline as the control or autologous blood (IVH). The white arrow indicates an expanded lateral ventricle. Data are shown as mean ± standard error of mean (SEM) (*n* = 5 in each group). *p < 0.05 and **p < 0.01 compared with saline group. **b** Representative T2*-weighted MRI scans (coronal brain sections) and quantification of T2* lesion volume at 7, 14, and 28 days after saline and blood injection. The red arrow indicates iron deposition. Values are mean ± SEM (*n* = 5 in each group). **p < 0.01 compared with saline group. **c** Representative Hematoxylin and eosin–stained sections showing ventricle wall damage on day 28 after intraventricular injection of saline and blood that are quantified in the scatter graph. The black arrow indicates a damaged ventricular wall. Values are mean ± SEM (*n* = 3 in each group). Scale bar = 50 µm. **p < 0.01 compared with saline group

Serial T2 and T2*-weighted MRI scans were performed to measure ventricular volume and iron deposition after intraventricular injection of autologous blood or saline. There was an increasing trend in lateral ventricular volumes in IVH rats compared with rats receiving saline injection beginning on day 7 (11.08 ± 1.77 vs. 6.51 ± 0.56 mm^3^ in the saline group, *p* > 0.05) (Fig. 1a) and reaching significance on day 14 (14.41 ± 1.13 vs. 6.68 ± 0.14 mm^3^, *p* < 0.01) (Fig. 1a) and day 28 (12.01 ± 1.84 vs. 6.01 ± 0.74 mm^3^, *p* < 0.05) (Fig. 1a). The volume of the intraventricular T2* hypointensity lesion, a measurement of iron deposition, was significantly higher in the IVH group than in the saline group from day 7 (2.63 ± 0.52 vs. 0.03 ± 0.03 mm^3^ in the saline group, *p* < 0.01) (Fig. 1b) to day 28 (1.25 ± 0.33 vs. 0.00 ± 0.00 mm^3^, *p* < 0.01) (Fig. 1b). There was a linear correlation between the volume of the intraventricular T2* hypointensity lesion and the volume of the lateral ventricle (*r* = 0.694, *p* < 0.001) (Supplementary Fig. 1).

Histological evaluation showed there was more severe ventricle wall damage and disruption of the ependymal surface in the IVH group compared with the saline injection group (53 ± 2% vs. 22 ± 4% in the saline group, *p* < 0.01) (Fig. 1c).

### IVH Causes an Upregulated Expression of AQP4 in the Periventricular Area

**Fig. 2:**
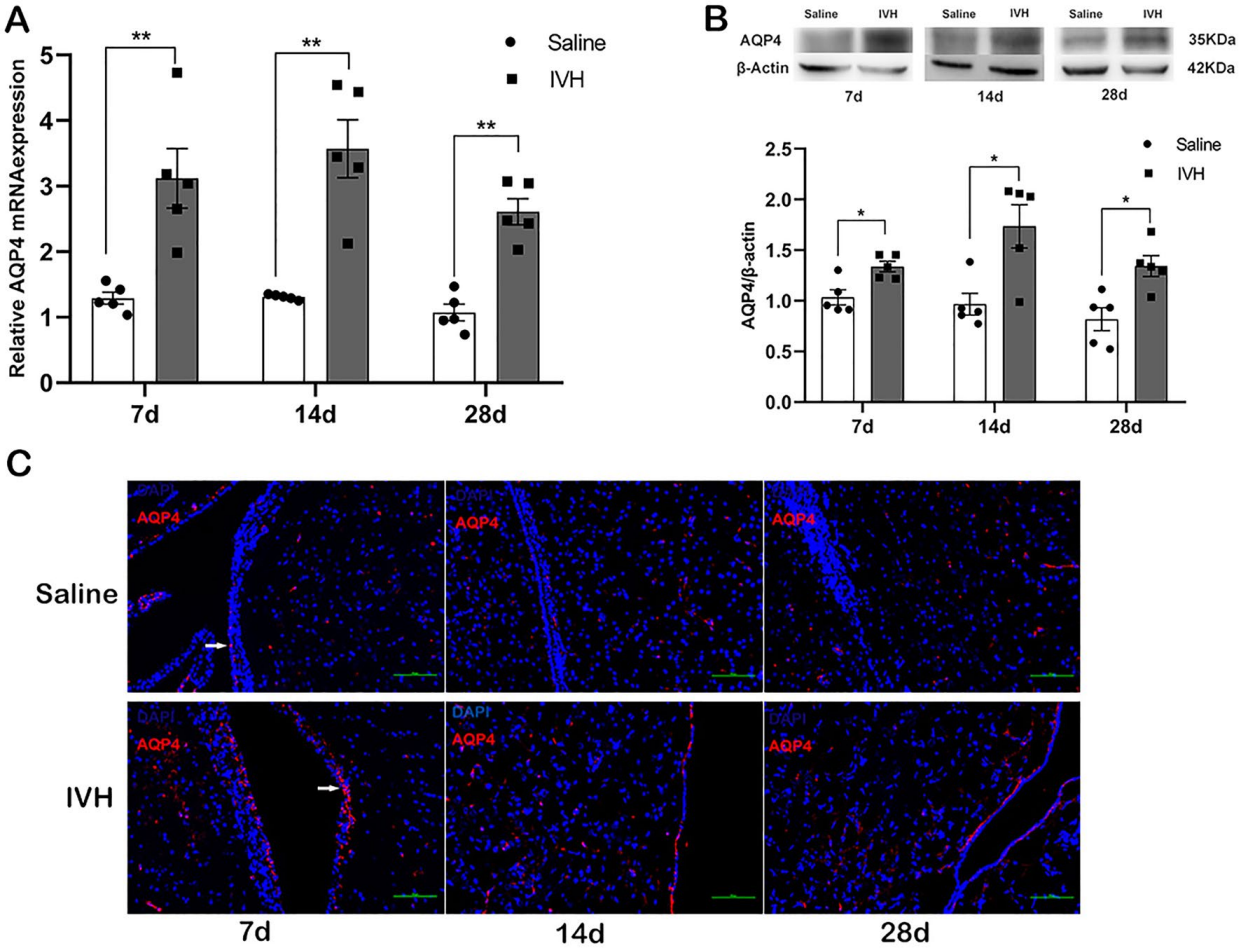
Intraventricular hemorrhage (IVH) causes an upregulated expression of aquaporin 4 (AQP4) in the periventricular area. mRNA (**a**) and protein (**b**) levels of AQP4 in the periventricular area of rats in the saline and IVH groups at 7, 14, and 28 days. β-actin acted as a control. **c**, Immunofluorescence staining of AQP4 in the periventricular area. The white arrow indicates AQP4 located on ependymal cells. Data are shown as mean ± standard error of mean (*n* = 5 per group). Scale bar = 100 µm. *p < 0.05 and **p < 0.01 compared with saline group

To investigate the expression of AQP4 after IVH, the levels of AQP4 mRNA and protein in the periventricular tissue were analyzed at 7, 14, and 28 days. There was a significant increase in AQP4 mRNA levels in the IVH group relative to the saline group at all time points (*p* < 0.01) (Fig. 2a). Similarly, AQP4 protein levels in the periventricular tissue were also higher in the IVH group than in the saline injection group from day 7 to day 28 (*p* < 0.05) (Fig. 2b). Further, immunofluorescence analysis showed that upregulated expression of AQP4 was mainly located on ependymal cells bordering the lateral ventricles (Fig. 2c).

### DFX Alleviates IVH-Induced Hydrocephalus, Iron Deposition, and Ventricular Wall Damage

**Fig. 3:**
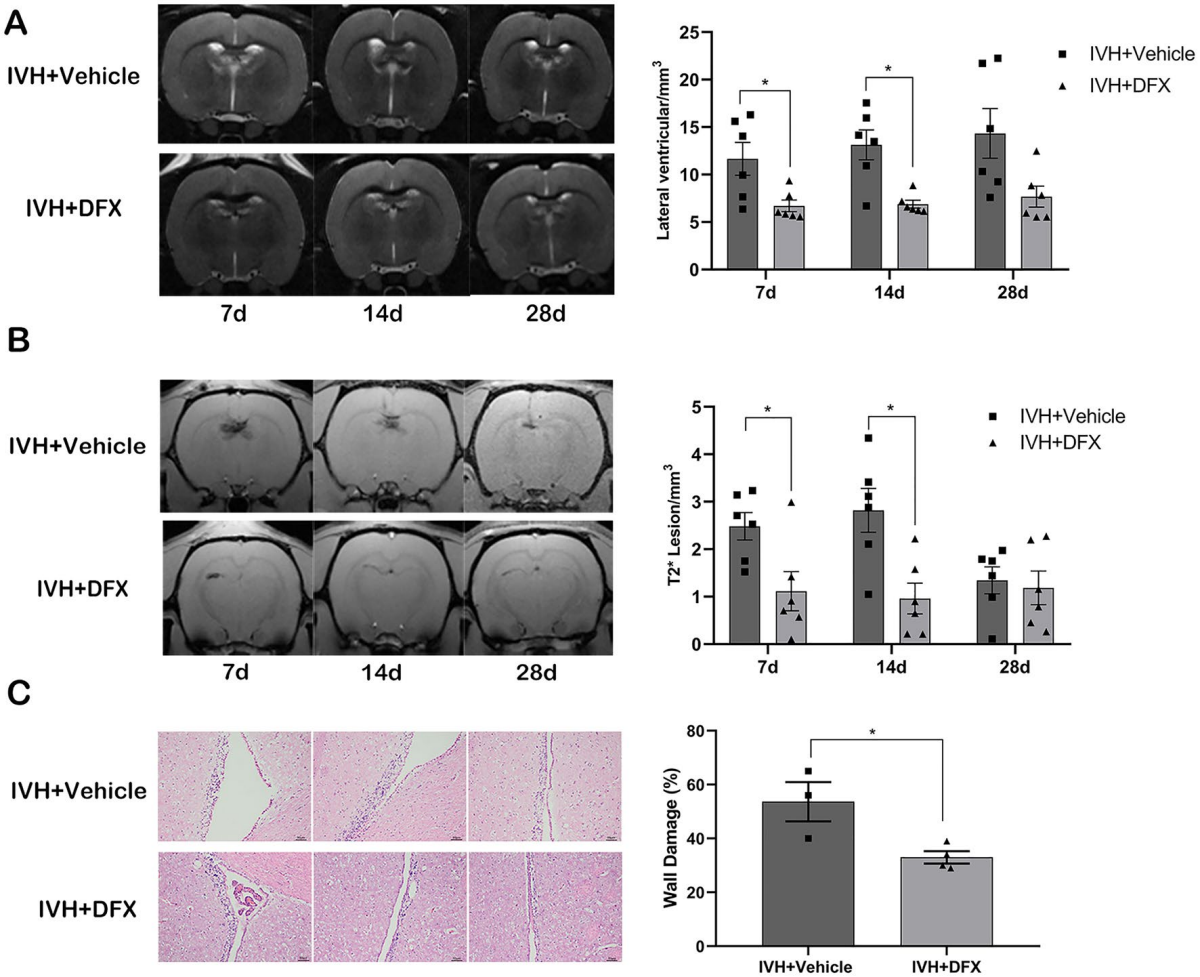
Deferoxamine (DFX) inhibits ventricular dilation, intraventricular iron deposition, and ventricular wall damage after intraventricular hemorrhage (IVH). **a** Representative T2-weighted magnetic resonance imaging (MRI) scans (coronal brain sections) at 7, 14, and 28 days after intraventricular injection of autologous blood in rats treated with vehicle or DFX and quantification of ventricular volume. Data are shown as mean ± standard error of mean (SEM) (*n* = 6 in each group). *p < 0.05 compared with IVH + vehicle group. **b** Representative T2*-weighted MRI scans (coronal brain sections) and quantification of T2* lesion volume at 7, 14, and 28 days after intraventricular injection of autologous blood in rats treated with vehicle or DFX. Values are mean ± SEM (*n* = 6 in each group). *p < 0.05 compared with IVH + vehicle group. **c**, Representative hematoxylin and eosin-stained sections showing ventricular wall damage and quantification of that damage. Values are mean ± SEM (*n* = 3–4 in each group). Scale bar = 50 µm. *p < 0.05 compared with IVH + vehicle group

DFX was used to test the effects of iron overload on ventricular dilatation after IVH. We observed a reduction of lateral ventricular volume in the DFX treatment group on day 7 (6.71 ± 0.62 vs. 11.65 ± 1.73 mm^3^ in vehicle controls, *p* < 0.05) (Fig. 3a) and day 14 (6.89 ± 0.42 vs. 13.13 ± 1.58 mm^3^, *p* < 0.05) (Fig. 3a), but there was no statistical difference on day 28 (7.69 ± 1.10 vs. 14.33 ± 2.62 mm^3^ in vehicle controls, *p* > 0.05) (Fig. 3a). Correspondingly, in the DFX-treated rats, the intraventricular T2* lesion volumes were also significantly lower than those in the vehicle-treated rats on day 7 (1.11 ± 0.41 vs. 2.48 ± 0.29 mm^3^ in vehicle controls, *p* < 0.05) (Fig. 3b) and day 14 (0.96 ± 0.33 vs. 2.82 ± 0.46mm^3^ in vehicle controls, *p* < 0.05) (Fig. 3b), but the difference became statistically insignificant on day 28 (1.19 ± 0.35 vs. 1.34 ± 0.28 mm^3^ in vehicle controls, *p* > 0.05) (Fig. 3b). Comparing with rats in the vehicle group, a significant reduction of ventricle wall damage was found in rats with DFX treatment on day 28 (33 ± 2% vs. 54 ± 7% in vehicle controls, *p* < 0.05) (Fig. 3c).

### DFX Inhibits the Expression of AQP4 in the Periventricular Tissue After IVH

**Fig. 4:**
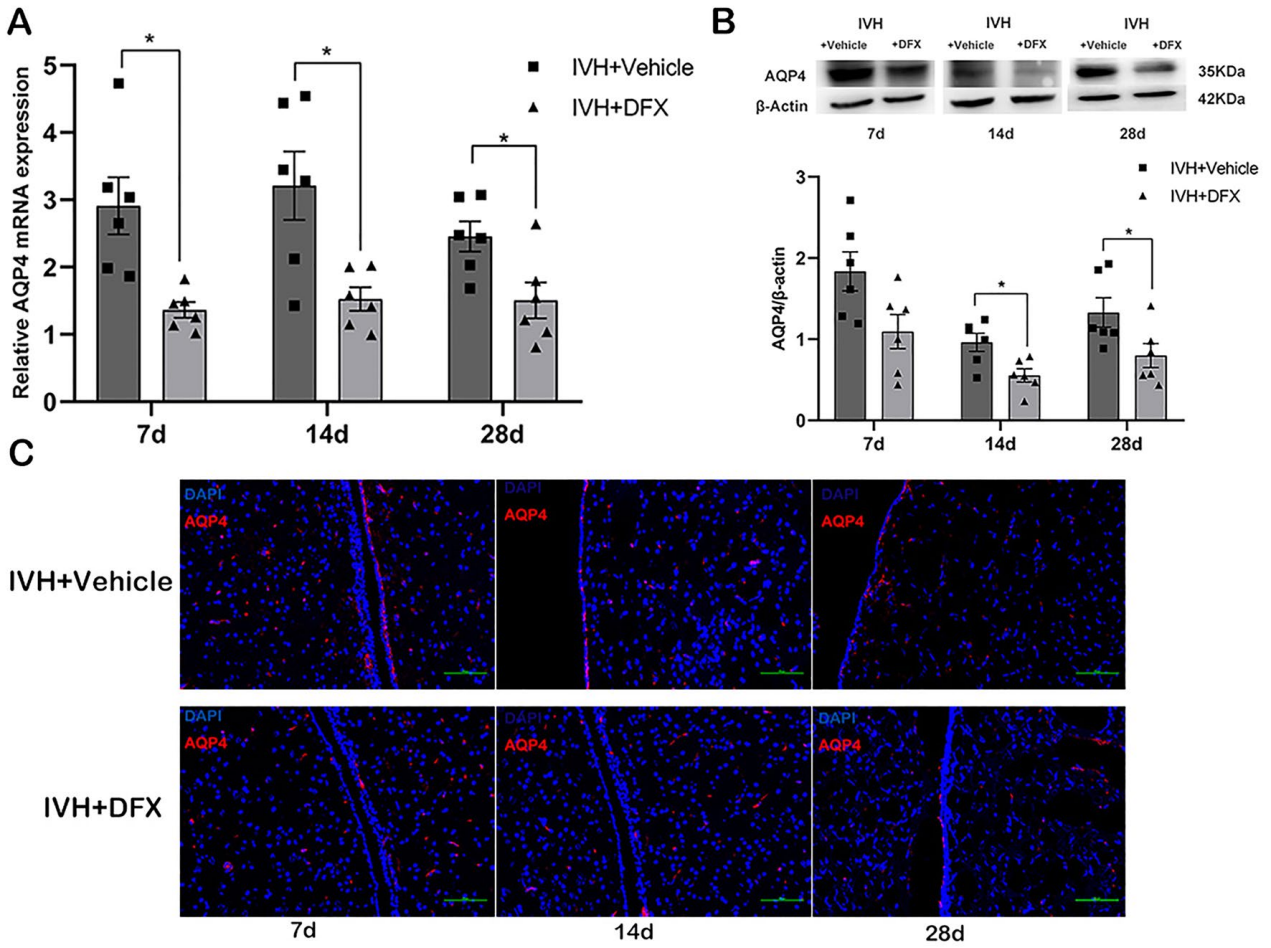
Deferoxamine (DFX) reduces the expression of aquaporin 4 (AQP4) after intraventricular hemorrhage (IVH). mRNA (**a**) and protein (**b**) levels of AQP4 in the periventricular area of rats treated with vehicle or DFX at 7, 14, and 28 days after intraventricular injection of autologous blood. β-actin was the control. **c** Immunofluorescence staining of AQP4 in the periventricular area. Data are shown as mean ± standard error of mean (*n* = 6 per group). Scale bar = 100 µm. *p < 0.05 and **p < 0.01 compared with IVH + vehicle group

To investigate the association between iron overload and AQP4 expression, the levels of AQP4 mRNA and protein expression in the periventricular tissue were analyzed in the DFX- and vehicle-treated rats. There was a significant reduction in the expression of AQP4 mRNA in the DFX-injected rats at all three time points after IVH compared with the vehicle-treated group (*p* < 0.05) (Fig. 4a). Similarly, the periventricular AQP4 protein levels in the DFX-treated rats were lower on days 14 and 28 (*p* < 0.05) (Fig. 4b). Immunofluorescence analysis showed that the expression of AQP4 protein in the periventricular area was inhibited in the DFX-treated group compared with the vehicle controls (Fig. 4c).

### TGN-020 Alleviates IVH-Induced Hydrocephalus

**Fig. 5:**
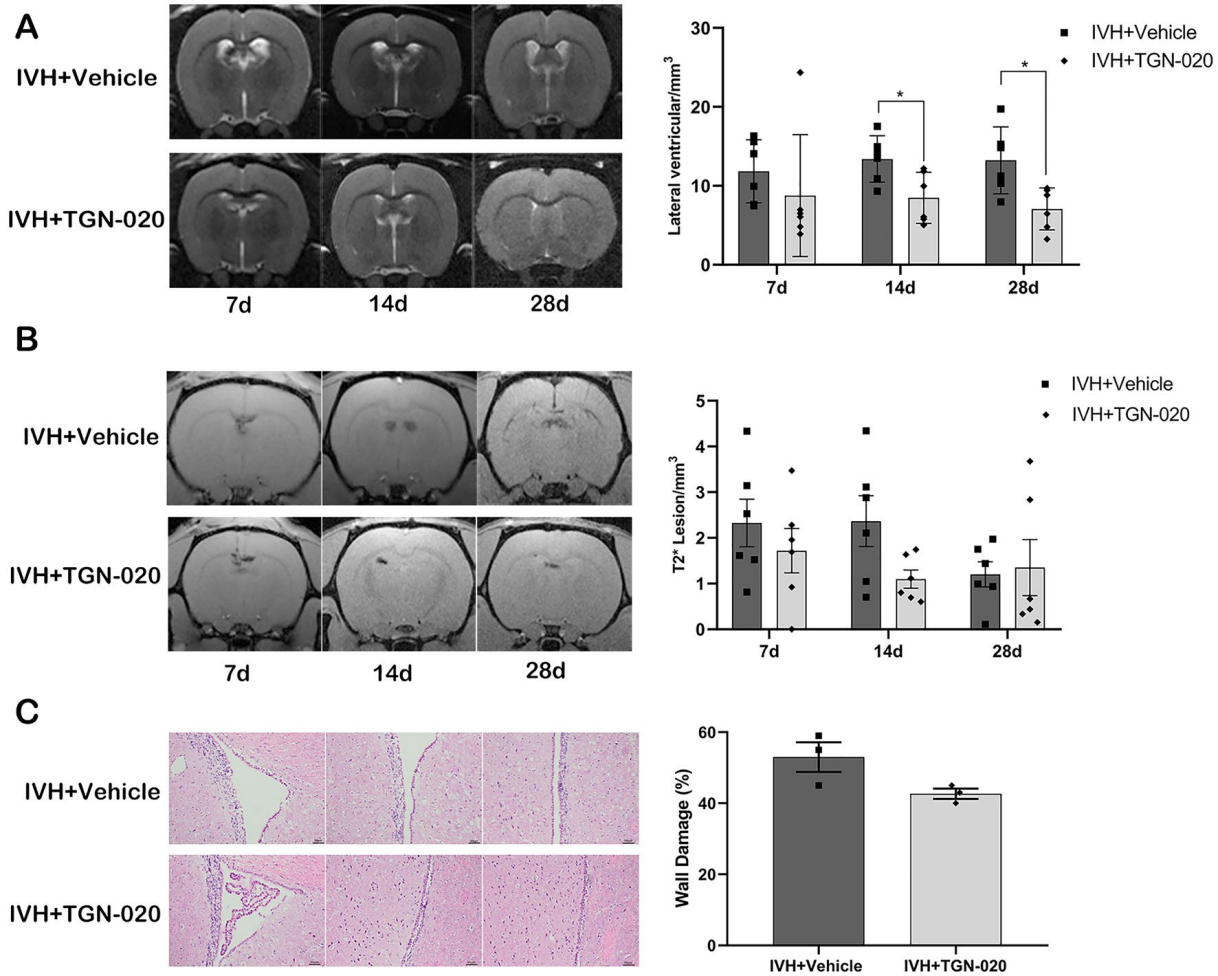
2-(nicotinamide)-1,3,4-thiadiazole (TGN-020) inhibits ventricular dilatation after intraventricular hemorrhage (IVH). **a** Representative T2-weighted magnetic resonance imaging (MRI) scans (coronal brain sections) at 7, 14, and 28 days after intraventricular injection of autologous blood in rats treated with vehicle or TGN-020 and quantification of ventricular volume. Data are shown as mean ± standard error of mean (SEM) (*n* = 6 in each group). *p < 0.05 compared with IVH + vehicle group. **b** Representative T2*-weighted MRI scans (coronal brain sections) and quantification of T2* lesion volume at 7, 14, and 28 days after intraventricular injection of autologous blood. Values are mean ± SEM (*n* = 6 in each group). **c** Representative hematoxylin and eosin–stained sections showing ventricular wall damage and quantification of that damage in the same two groups. Values are mean ± SEM (*n* = 3 in each group). Scale bar = 50 µm

TGN-020 was used to test the association between AQP4 and hydrocephalus and iron deposition after IVH. There was a trend of decreasing lateral ventricular volumes in TGN-020-treated rats compared with vehicle-treated rats on day 7 after IVH (8.77 ± 3.15 vs. 11.84 ± 1.63 mm^3^ in vehicle controls, *p* > 0.05) (Fig. 5a), and the difference reached significance at day 14 (8.48 ± 1.32 vs. 13.40 ± 1.20 mm^3^ in vehicle controls, *p* < 0.05) (Fig. 5a) and day 28 (7.07 ± 1.09 vs. 13.22 ± 1.73 mm^3^ in vehicle treatment group, *p* < 0.05) (Fig. 5a). The intraventricular T2* lesion volume between the TGN-020 treatment group and the vehicle control group did not differ significantly at any of the three time points (*p* > 0.05) (Fig. 5b). And the difference in the degree of ventricular wall damage was not statistically significant between the TGN-020 group and the vehicle group either (43 ± 1% vs. 53 ± 4% in vehicle controls, *p* > 0.05) (Fig. 5c).

### TGN-020 Inhibits the Expression of AQP4 in the Periventricular Tissue After IVH

**Fig. 6:**
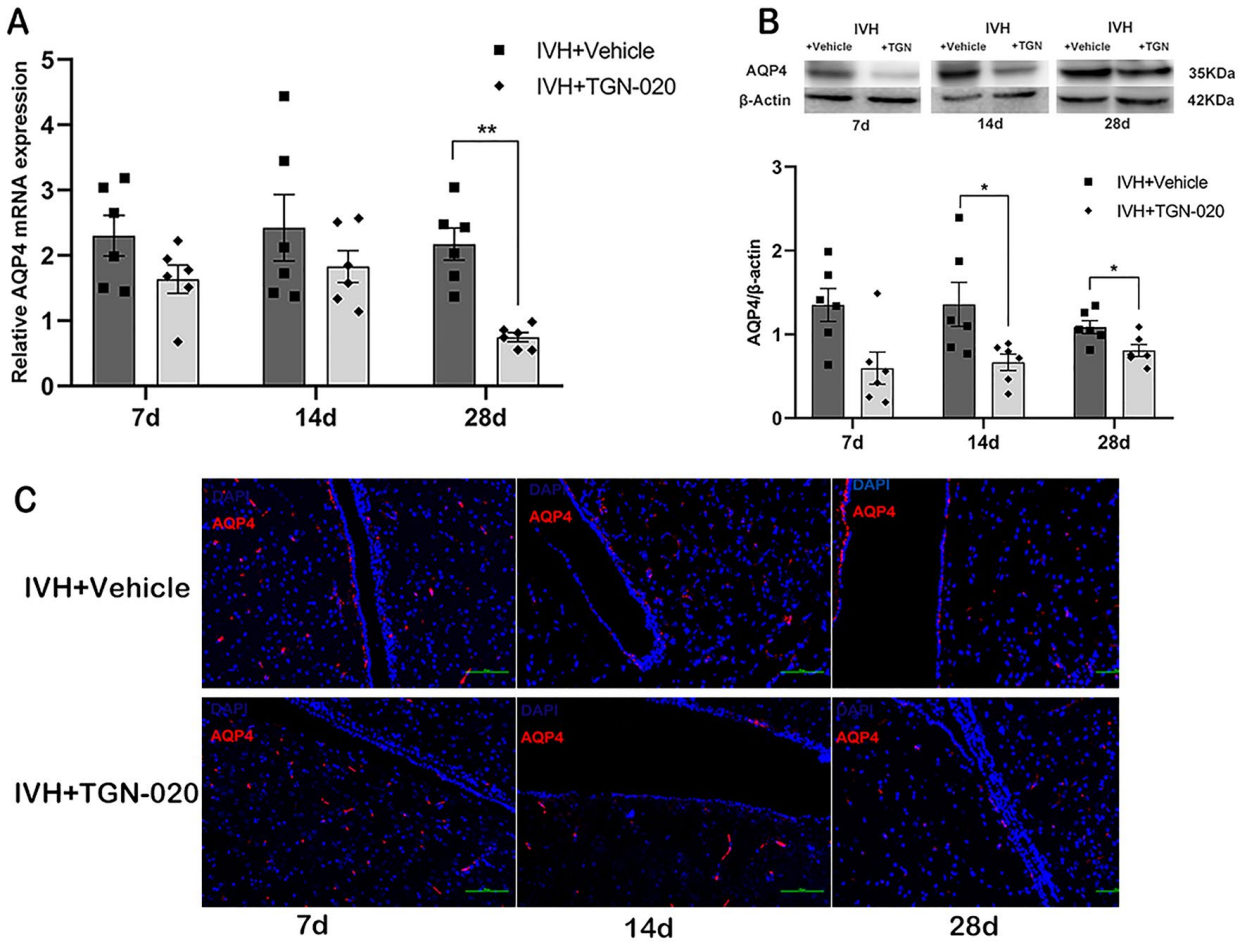
2-(nicotinamide)-1,3,4-thiadiazole (TGN-020) reduces the expression of aquaporin 4 (AQP4) after intraventricular hemorrhage (IVH). mRNA (**a**) and protein (**b**) levels of AQP4 in the periventricular area of rats treated with vehicle or TGN-020 at 7, 14, and 28 days after intraventricular injection of autologous blood. β-actin was the control. **c** Immunofluorescence staining of AQP4 in the periventricular area. Data are shown as mean ± standard error of mean (*n* = 6 per group). Scale bar = 100 µm. *p < 0.05 and **p < 0.01 compared with IVH + vehicle group

The levels of AQP4 mRNA in the periventricular tissue decreased in the TGN-020-treated group on day 28 (*p* < 0.01) (Fig. 6a). There was a significant reduction in the expression of AQP4 protein in the TGN-020-treated group on day 14 and day 28 (*p* < 0.05) (Fig. 6b). Immunofluorescence analysis also showed a reduction of AQP4 expression in the periventricular area (Fig. 6c).

## Discussion

**Fig. 7:**
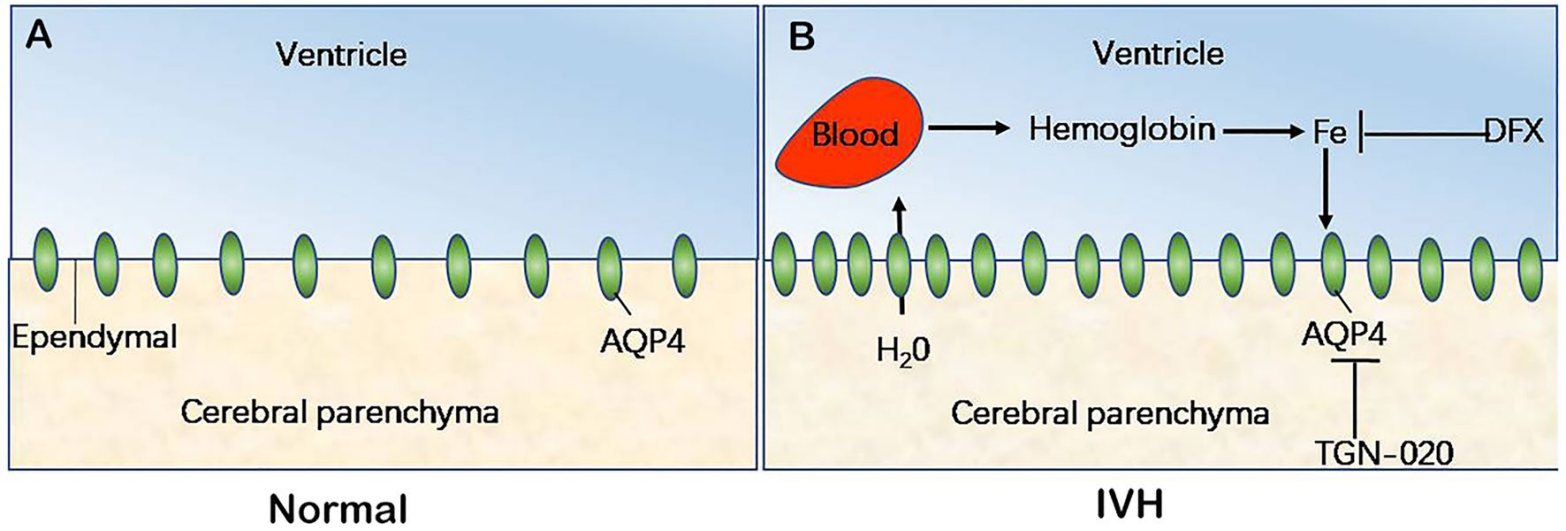
Schematic diagram of the role of aquaporin 4 (AQP4) in the formation of hydrocephalus after intraventricular hemorrhage (IVH). The model illustrates AQP4 expression in ependymal cells under normal physiological conditions (**a**) and after IVH (**b**). After IVH, red blood cells ruptured and hemoglobin was released. Iron, the degradation product, could increase the expression of AQP4 in ependymal cells, leading to interstitial fluid moving into the ventricles and the formation of hydrocephalus

The major findings in the present study were (1) intraventricular injection of autologous blood caused ventricular dilatation, iron deposition, and a significantly upregulated expression of AQP4 in the periventricular tissue; (2) DFX inhibited hydrocephalus development and iron deposition as well as the upregulated expression of AQP4 in the periventricular tissue after IVH; and (3) TGN-020 attenuated ventricular dilatation and the expression of AQP4 following IVH without a significant effect on intraventricular iron deposition or ventricular wall damage. Our results showed, for the first time, that AQP4 in the periventricular tissue contributed to the development of hydrocephalus caused by iron overload after IVH (Fig. 7).

Our study showed an increased expression of AQP4 in the periventricular area from day 7 to day 28 in hydrocephalus rats after IVH, which was consistent with previous literatures [15]. Several studies reported an increase in the expression of AQP4 in rat models with inherited and kaolin-induced hydrocephalus [19, 20]. In addition, a higher level of AQP4 in ependymal cells was found in rats with hydrocephalus than in rats without hydrocephalus in the model of SAH, and the expression of AQP4 remarkably correlated with the severity of hydrocephalus [16]. Schmidt et al. [21] reported that the concentrations of AQP4 increased in the CSF of dogs with idiopathic communicating internal hydrocephalus. In humans, cortical brain biopsy samples from patients with chronic hydrocephalus also revealed increased AQP4 expression [22].

Further, in our study, blocking AQP4 by injection of TGN-020 alleviated ventricular dilatation after IVH, which indicated that AQP4 contributed to the development of hydrocephalus. Another study on hypoxia-induced hydrocephalus also supported a promoting role of AQP4 in the development of hydrocephalus, finding chronic hypoxia to be associated with an increase in the total ventricular volume in wild-type mice, whereas the degree of ventricular dilatation was reduced in AQP4^−/−^ mice [23]. A recent study reported that repetitive hypoxic events in aged cerebral tissue promoted a permanent ventriculomegaly, which was dependent on AQP4 expression [24].

Additionally, there is increasing evidence that AQP4 expressed in ependymal cells and glia limitans is involved in the formation of CSF independent of choroid plexus secretion in physiological conditions [24–28]. One study found that AQP4^−/−^ mice showed a comparable reduction in ventricular volume and intraventricular pressure compared with AQP1^−/−^ mice, suggesting that AQP4 and AQP1 had a similar contribution to the production of CSF [25]. Using a water molecular imaging technique, Igarashi et al. [26] found a significant reduction in water influx into the CSF space in AQP4^−/−^ mice following intravenous injection of H_2_^17^O, whereas the behavior of water molecules in AQP1^−/−^ mice was virtually identical to that in wild-type mice. These results indicate a critical role of AQP4 in water movement into the ventricular system and subarachnoid space.

The findings in these studies suggested that the upregulated expression of AQP4 in the periventricular tissue prompted water movement into the ventricles and the formation of hydrocephalus after experimental IVH. However, some researchers thought the increased AQP4 expression associated with hydrocephalus represented a compensatory response to increase brain water clearance and reduce ventricular enlargement [19, 20]. In kaolin-injection-induced models of acute obstructive hydrocephalus, AQP4^−/−^ mice developed greater ventriculomegaly, developed higher intracranial pressure, and had worse outcomes compared with the wild-type mice [20].

We speculated that the disparity in the role of AQP4 might originate from different mechanisms of hydrocephalus development. Since AQP4 is a bidirectional water channel, the direction of flux through AQP4 is mainly dependent on the osmolarity difference between the two spaces connected by AQP4 [11, 12]. In our study, intraventricular injection of autologous blood caused an increase in crystal and colloidal osmotic pressure in the ventricle. Then the increased expression of AQP4 in the ependymal cells facilitated osmotically driven water movement from the brain parenchyma into the ventricle and the development of hydrocephalus. Blocking AQP4 with TGN-020 inhibited flux into the ventricle and alleviated ventricular dilation. While in the obstructive hydrocephalus model induced by kaolin injection, an elevated intraventricular hydrostatic pressure drove water movement from the ventricle into the parenchyma through AQP4 located on ependymal cells, presenting a compensatory effect for hydrocephalus.

Consistent with previous studies, we found extensive iron deposition in the lateral ventricle after intraventricular injection of autologous blood, which originated from hemoglobin degradation [5, 6, 29]. We also found a linear correlation between iron deposition and lateral ventricle volume, and DFX could inhibit ventricular dilatation after IVH, which supported a key role of iron in the development of hydrocephalus after IVH [6, 29, 30]. Additionally, we observed that DFX could inhibit the upregulation of AQP4 expression in the periventricular tissue after IVH. A similar phenomenon was reported by Qing et al. [31] in a rat model of ICH. They found that iron deposition around the hematoma was accompanied by upregulation of AQP4 expression, and DFX treatment significantly reduced iron deposition and AQP4 expression in the brain tissue around the hematoma [31]. Further, the inhibition of AQP4 by injection of TGN-020 did not affect intraventricular iron accumulation in our study. Taken together, these results imply that iron overload should be the cause of the change in the expression of AQP4 after IVH, not the reverse.

Although many studies indicated a critical role of iron overload in the development of hydrocephalus after IVH, there was limited information regarding the molecular mechanism. Based on observations of the expression of Wnt1/Wnt3a mRNA and protein levels in IVH rats treated with DFX, some scholars speculated that iron might promote the subarachnoid fibrosis and hydrocephalus after IVH by activation of the Wnt signaling pathway [5]. But the association between the Wnt pathway and hydrocephalus has not been confirmed. We found that iron overload could affect the expression of AQP4 in the periventricular area, and the upregulated expression of AQP4 participated in the development of hydrocephalus after IVH. Considering AQP4 is the main water channel protein in the brain, our results suggested that AQP4 may mediate the effect of iron overload on hydrocephalus after IVH, which not only contributes to the understanding of the mechanism of iron-induced hydrocephalus but also provides a potential therapeutic target for hydrocephalus after IVH.

As a specific inhibitor of AQP4, TGN-020 has been reported to bind to AQP4 directly and play a pharmacological blockade role [14, 32, 33]. In our study, TGN-020 inhibited the expression of AQP4 protein on days 14 and 28 after IVH and could even reduce the expression of AQP4 mRNA after 28 days of continuous application. Previous studies showed that TGN-020 inhibited the expression of AQP4 protein in the spinal cord injury model, crushed optic nerves model, and diabetic retina model [17, 34, 35]. Another study found that TGN-020 could reduce the expression of AQP4 mRNA and protein in A549 cells exposed to silicon dioxide [36]. Therefore, some scholars suggested defining TGN-020 as an AQP4 modulator rather than an AQP4 inhibitor [37]. The dual effects on AQP4 indicate that TGN-020 is a promising agent for the treatment of hydrocephalus after IVH.

In our study, we found that more severe ventricle wall damage after IVH and DFX could alleviate the change, which was consistent with a previous report [38]. Although TGN-020 did not show a significant benefit in the protection of the ventricle wall, this finding indicated that a decreased ventricular volume after IVH in the TGN-020-treated group might have contributed to the inhibition of AQP4 and CSF secretion by TGN-020 rather than a reduction in periventricular injury. It supported our research hypothesis that AQP4 took a role in the development of hydrocephalus after IVH.

There are several limitations to this study. First, recent studies have shown that in addition to iron, other degradation products of hemoglobin (e.g., peroxiredoxin 2) also contribute to the development of hydrocephalus after IVH [18]. The role of AQP4 in these pathways should be explored in further research. Second, we found an increase in AQP4 expression in response to iron overload. Several studies have suggested that the mitogen-activated protein kinases and nuclear factor κB p65 pathways may contribute to this process [11, 39, 40], but the exact mechanism remains to be determined. Third, although some studies observed water movement into the ventricular system dependent on AQP4, some scholars thought the available evidence could not confirm the direction of the net fluid movement through AQP4 and questioned the role of AQP4 in the formation of CSF in physiological conditions [41, 42]. Further studies on the net flux dependent on AQP4 are needed to strengthen our understanding of the role of AQP4 after IVH. Fourth, although TGN-020 was used as a specific inhibitor of AQP4 in previous studies, some articles reported it may have an affinity for AQP1 in vitro [32]. Further study with the simultaneous evaluation of AQP1 and AQP4 expression will be helpful to confirm the role of AQP4 in the development of hydrocephalus after IVH. Fifth, pentobarbital was used as the anesthetic drug in our study, which may affect body temperature and blood pressure of the animal models. We used a feedback heated blanket to maintain core body temperature of the rats. But we did not monitor blood pressure of rats during anesthesia. Considering all rats received pentobarbital in our experiment, we speculated that the use of pentobarbital had a limited influence on the evaluation of the role of AQP4 in hydrocephalus. In a future study, a comprehensive monitoring of physiological variables, including intracranial pressure and blood pressure, may improve the strength of our results. Finally, we observed the inhibiting effect of TGN-020 on hydrocephalus after IVH through MRI examination. Behavioral tests are essential to evaluate the clinical potential of TGN-020 as a treatment for IVH.

## Conclusions

In conclusion, the present study revealed that iron overload causes an upregulation of AQP4 expression in the periventricular area, which further contributes to the development of hydrocephalus after IVH. AQP4 may be a potential therapeutic target for hydrocephalus after IVH.

### Supplementary Information

Below is the link to the electronic supplementary material.

Supplementary file1 (TIF 332 kb)

## Data Availability

All data generated/analyzed during this study are included in this published article. All other relevant data that support the findings of this study are available from the corresponding author upon reasonable request.
