# Supplementary figures and images for: Aquaporin 4 Mediates the Effect of Iron Overload on Hydrocephalus After Intraventricular Hemorrhage
Source: Neurocrit Care. 2023 May 19;40(1):225–36. doi: 10.1007/s12028-023-01746-w (PMC10861395; doi:10.1007/s12028-023-01746-w)

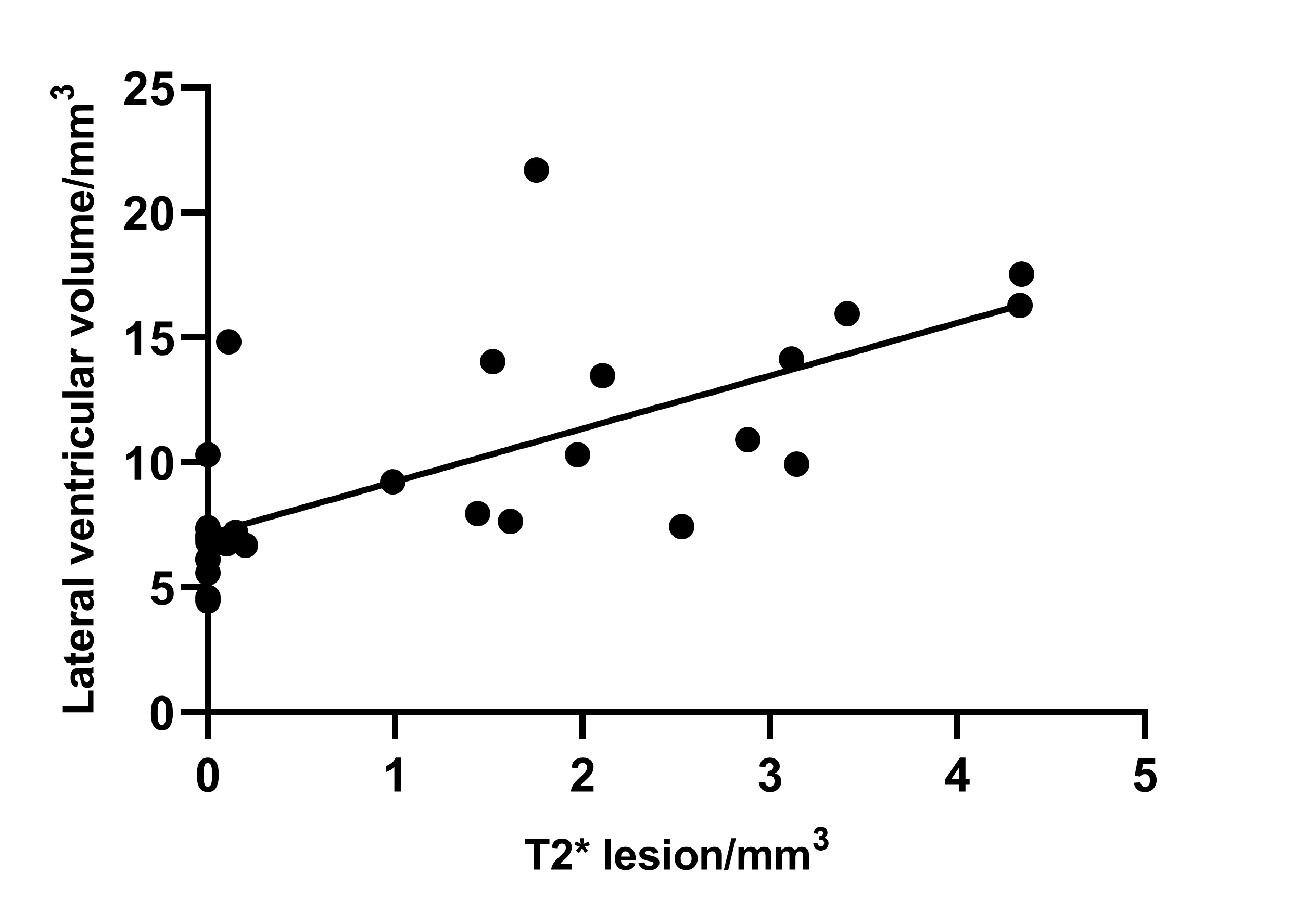

Supplement: Supplementary file 1 — Supplementary file1 (TIF 332 kb) [file 12028_2023_1746_MOESM1_ESM.tif]
